# The effect of Fe_2_O_3_ crystal phases on CO_2_ hydrogenation

**DOI:** 10.1371/journal.pone.0182955

**Published:** 2017-08-14

**Authors:** Wensheng Ning, Tianqi Wang, Hongxian Chen, Xiazhen Yang, Yangfu Jin

**Affiliations:** 1 College of Chemical Engineering, Zhejiang University of Technology, Hangzhou, China; 2 College of Materials Science and Technology, Zhejiang University of Technology, Hangzhou, China; Institute of Materials Science, GERMANY

## Abstract

The effect of Fe_2_O_3_ crystal phases on their performance in CO_2_ hydrogenation was studied. α-Fe_2_O_3_ crystal was prepared by precipitation method from Fe(NO_3_)_3_·9H_2_O and (NH_4_)_2_CO_3_, and γ-Fe_2_O_3_ was prepared by grinding Fe(NO_3_)_3_·9H_2_O and L(+)-Tartaric acid in agate mortar completely. The crystal phases of Fe_2_O_3_ influence the distribution of promoter Zn, K and Cu on catalysts. The dispersity of K on γ-Fe_2_O_3_ surface is higher than α-Fe_2_O_3_. On the contrary, Cu and Zn are more dispersive on α-Fe_2_O_3_ surface than γ-Fe_2_O_3_. The catalyst in γ-Fe_2_O_3_ phase is easily reduced relative to the catalyst in α-Fe_2_O_3_ phase. The former presents higher CO_2_ conversion and C_2_+ hydrocarbon selectivity than the latter in CO_2_ hydrogenation.

## Introduction

CO_2_ hydrogenation for organic chemicals is a worthy study under the background that CO_2_ used as raw material for chemicals other than discharged into atmosphere would be helpful to abate the greenhouse effect. However, the difficulty to capture CO_2_ and the cost to supply H_2_ make most of the researches stayed in laboratory. The concept and trial using seawater as starting materials brings an applicable and profitable scene to CO_2_ hydrogenation [1–4]. Seawater is a natural absorbent of CO_2_, from which plenty of CO_2_ can be captured. Seawater is an unlimited source for H_2_, too. The above concept becomes accepted to us because the device to transfer solar power into electricity can be constructed on the vast ocean, which would supply enough energy to produce CO_2_ and H_2_ from seawater simultaneously. With the concept breakthrough where and how to perform CO_2_ hydrogenation, active catalysts are the key component to commercialize CO_2_ hydrogenation.

The organic chemicals synthesized from CO_2_ hydrogenation include methane, methanol, methyl acid, dimethyl ether, hydrocarbons and mixed alcohols [5–7]. Among them, hydrocarbons are a good product because it can be upgraded into liquid fuels which are cleaner than the petroleum-based fuels [8]. It is accepted that CO_2_ is hydrogenated into hydrocarbons by two steps: CO is produced from CO_2_ by reverse Water-Gas shift (WGS) reaction (Reaction I), then the CO reacts with H_2_ to synthesize hydrocarbons via Fischer-Tropsch synthesis (FTS) (Reaction II) [9–14].

$${\text{Reaction I: CO}}_{\text{2}}{\;+\text{ H}}_{\text{2}}{\text{ = CO }+\text{ H}}_{\text{2}}\text{O}$$

$$\text{Reaction II: CO }+{\text{ 2H}}_{\text{2}}=\;\left({\text{CH}}_{\text{2}}\right)\;+{\text{ H}}_{\text{2}}\text{O}$$

Fe and Co are commercial catalysts for FTS. Riedel et al. [15] compared the performance of Fe and Co catalysts in the mixtures of CO, CO_2_, and H_2_. With increased CO_2_ and decreased CO content in the feedgas, the product composition shifted from a mixture of mainly higher hydrocarbons to almost exclusively methane for Co catalyst, while Fe catalyst synthesized the same hydrocarbon products from CO_2_ /H_2_ as from CO/H_2_ syngas. Zhang et al. [16] also found that the CO_2_ hydrogenation products contained about 70% or more methane for supported Co catalyst. These distinctions are partly attributed to that Fe catalyst is active for both of the Reaction I and II [15,17,18].

In order to improve the performance of Fe catalysts in CO_2_ hydrogenation, the effects of promoter [8,10,13,15,19–24], supporter [15,20,22,24,25], preparation method [8,13,15,21–26] and reducing agent [8] are studied very much. In these studies, iron oxide almost presents in α-Fe_2_O_3_ [15,21,26] or Fe_3_O_4_ [25] crystal phase in the as-prepared catalysts. Considering that γ-Fe_2_O_3_ is one kind of iron oxide as common as α-Fe_2_O_3_ [27,28], it is surprising that there are very few reports about the behavior of γ-Fe_2_O_3_ in CO_2_ hydrogenation. Al-Dossary et al. found γ-Fe_2_O_3_ coexisted with α-Fe_2_O_3_ in the catalysts, but no benefit from γ-Fe_2_O_3_ was disclosed [21]. However, it has been confirmed that γ-Fe_2_O_3_ is superior to α-Fe_2_O_3_ in other catalytic reactions, such as photodecomposition of H_2_S [29], selective catalytic reduction of NO_X_ with NH_3_ [30], electroanalysis and ultrasensitive detection of Pb^2+^ [27], WGS reaction [31] and so on. The lack on the performance of γ-Fe_2_O_3_ in CO_2_ hydrogenation makes it necessary to study Fe catalyst in γ-Fe_2_O_3_ phase, not only to supply the knowledge about γ-Fe_2_O_3_ in the reaction, but also to find active catalyst to make CO_2_ profitable.

We has reported the influences of Fe_2_O_3_ crystal phases on CO_2_ hydrogenation [32]. γ-Fe_2_O_3_ phase in the catalysts was formed by washing FeAl precipitate with anhydrous ethanol. The catalyst with strong γ-Fe_2_O_3_ phase was more active in the reaction than the catalysts with none or weak γ-Fe_2_O_3_ phase. In order to avoid the possible promotion of Al on the catalyst activity and prepare the catalyst in pure γ-Fe_2_O_3_ phase simultaneously, solid-phase reaction was used recently for catalyst preparation. The effect of Fe_2_O_3_ phase on the catalyst reactivity is explored in this work.

## Materials and methods

### Catalyst preparation

Three kinds of catalyst precursor were prepared. P-1 was prepared by precipitating Fe(NO_3_)_3_·9H_2_O solution with (NH_4_)_2_CO_3_ solution under vigorous stirring at 50°C and pH = 6.5. The resulting precipitate was aged at 50°C for 0.5 h and room temperature for 1 h. After it was washed with distilled water and centrifuged for three times, the precipitate was dried at 120°C overnight and calcined at 500°C for 6 h in static air [32]. P-2 was prepared by grinding Fe(NO_3_)_3_·9H_2_O and L(+)-Tartaric acid (1:1 in mass ratio) in agate mortar completely. The obtained deep red solid was washed with dehydrated alcohol for three times. Then, the solid was dried at 80°C for 3 h and calcined at 400°C for 1 h. P-3 was prepared in the same procedure as P-2 except some water was added during the grinding. The precursors were shaped into particles of 150–280 μm and impregnated with Zn, K and Cu in the mass ratio of 2%, 3% and 4%, respectively. After the impregnated precursors were dried at 120°C for 12 h, the impregnated P-1 was calcined at 500°C for 6 h, while the impregnated P-2 and P-3 were calcined at 400°C for 1 h. The promoted catalysts were named as C-1, C-2 and C-3 correspondingly.

### Characterization

The crystal structure of the catalysts was acquired by X-ray diffraction (XRD, PNAlytical X’Pert Pro diffractometer) with a Cu K_α_ radiation source (λ = 0.15406 nm) in reflection mode. X-ray tube was operated at 40 kV and 40 mA. Surface area and pore structure of the samples were measured by ASAP-2020 from Micromeritics at liquid nitrogen temperature. Temperature-programmed reduction (TPR) was carried out in PX200 (Tianjin Pengxiang LTD.) with 5% H_2_/N_2_ of 30 mL/min and a TCD detector. The sample was heated to 850°C at the rate of 10°C/min. The morphology of the catalysts was observed by scanning electron microscopy (SEM, Hitachi S-4700II) which was attached with an energy dispersive spectroscopy (EDS, Thermo NORAN VANTAGE ESI.).The accelerating voltage is 15 kV. The results from SEM and EDS are shown in S1 and S2 Figs, respectively. XPS analysis was done at Catalysis and Surface Science End-station of National Synchrotron Radiation Laboratory in University of Science & Technology of China using Mg K_α_ radiation (1253.6 eV) and VG SCIENTA R4000 analyzer. The binding energy of C 1s (285.0 eV) was used to calibrate the peak position of other elements.

### Activity test and product analysis

The reactivity of catalysts was tested in a stainless steel fixed bed reactor of inter-diameter of 8 mm [19]. A 1.0 g catalyst (150–280 μm) was mixed with 4.0 g quartz sand and they were filled into the reactor. After the catalyst was reduced in CO of 3.0 L/(h·g-cat) at 300°C for 6 h, it was cooled to room temperature. Then, the feed gas was changed into mixed gases of H_2_:CO_2_:N_2_ = 16:8:1 of 1.6 MPa and 6.0 L/(h·g-cat). The catalyst was heated to 230°C in about 3 h for activity evaluation of 45 h. The condensable products were collected in a cold trap of 0°C at system pressure. After the system pressure was released through a backpressure regulator, the exited gas was analyzed by GC A90 (Shanghai Yimeng LTD.) on line. The quantities of CO, CH_4_, CO_2_ and N_2_ were supplied with TCD detector and TDX-01 column. C_1_–C_4_ hydrocarbons were analyzed with FID detector and Porapak Q column.

## Results and discussion

### Crystal phase of the precursors and catalysts

**Fig 1:**
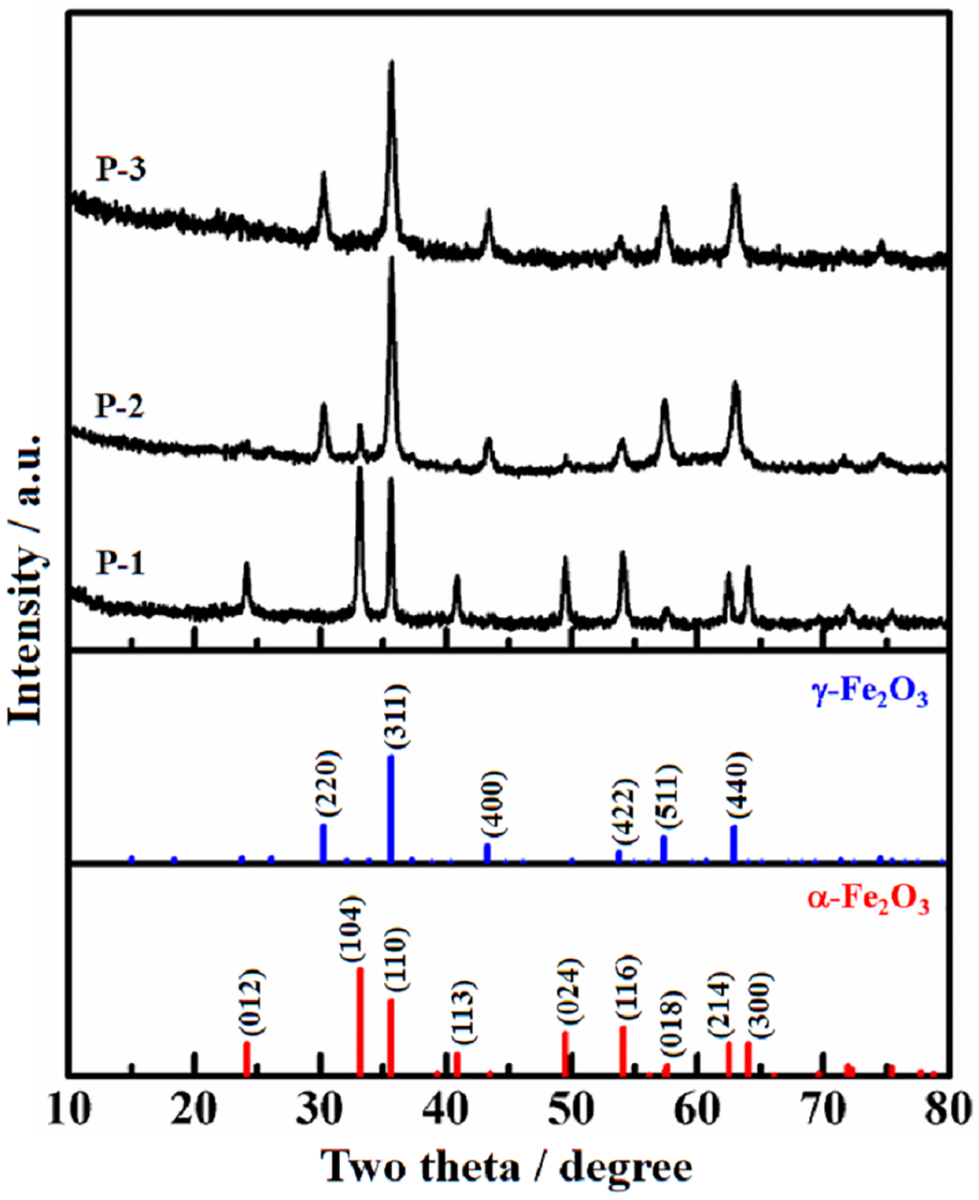
XRD patterns of the precursors, as well as the standard data for α-Fe_2_O_3_ and γ-Fe_2_O_3_.

Fig 1 shows the XRD patterns of precursor P-1, P-2 and P-3. There is only α-Fe_2_O_3_ (PDF: 33–0664) detected in P-1, and only γ-Fe_2_O_3_ (PDF: 39–1346) in P-3, while P-2 contains α-Fe_2_O_3_ and γ-Fe_2_O_3_ phases simultaneously. Calculated with Scherer equation [33] based on 35.6° peak, the particle size is 32.2 nm (P-1), 17.7 nm (P-2) and 16.3 nm (P-3), respectively. The data indicates that the particle size in the precursor built by γ-Fe_2_O_3_ is small.

**Fig 2:**
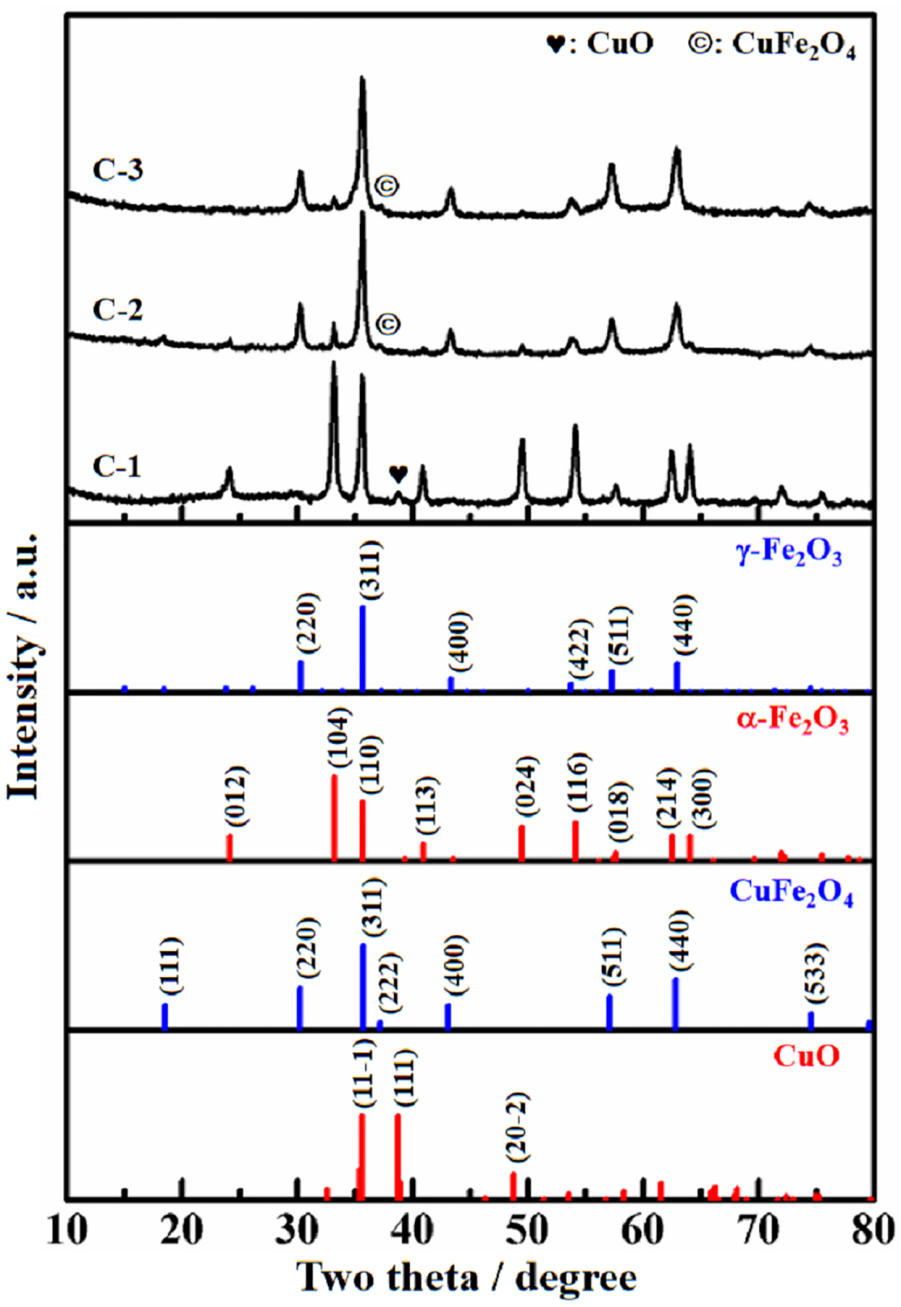
XRD patterns of the catalysts, as well as the standard data for CuO, CuFe_2_O_4_, α-Fe_2_O_3_ and γ-Fe_2_O_3_.

Fig 2 is the XRD patterns of catalyst C-1, C-2 and C-3. The Fe_2_O_3_ phases in them are the same as their precursors. The different crystal phases of Fe_2_O_3_ influence the distribution of promoter Zn, K and Cu in C-1, C-2 and C-3. CuO (PDF: 48–1548) is found in C-1, while CuFe_2_O_4_ (PDF: 25–0283) appears in C-2 and C-3. The dispersion of Cu in C-1 is lower than C-2 and C-3. The existing state of Cu is related to the particle size of Fe_2_O_3_ in the precursors. Because the Fe_2_O_3_ size of P-2 and P-3 is only about half of P-1, the dispersed degree of iron atom in P-2 and P-3 is much higher than P-1. The impregnated copper atom can contact with more iron atom in P-2 and P-3 than P-1, which is able to explain why CuFe_2_O_4_ was formed in C-2 and C-3. Although the crystal containing Zn and (or) K is not detected in Fig 2, the existence of Zn and K in the catalysts is confirmed by XPS analysis.

### Texture of the precursors and catalysts

Table 1 lists the BET surface area and pore distribution of the precursors and catalysts measured by N_2_-adsorption at liquid nitrogen temperature. For the precursors, the specific surface area of P-2 and P-3 is bigger than P-1, which can partly explained by the particle size of Fe_2_O_3_ in them. Small particle usually constitutes a collective with large surface area. However, the specific surface area of P-3 is less than P-2 regardless of the particle size in P-3 (16.3 nm) is smaller than P-2 (17.7 nm). This contradiction can be solved by the fact that the theoretical densities of γ-Fe_2_O_3_ (5.47 g·cm^-3^) is higher than α-Fe_2_O_3_ (5.27 g·cm^-3^) [28]. According to Fig 1, P-2 is a mixture of α-Fe_2_O_3_ and γ-Fe_2_O_3_, while P-3 is composed of pure γ-Fe_2_O_3_. As a result, the volume per unit mass of P-2 is larger than P-3. It means that P-2 is in looser state than P-3. Both of the average pore diameter and pore volume are in the order of P-1 > P-2 > P-3.

**Table 1:** Texture of the precursors and catalysts.

Sample	BET surface area (m^2^/g)	Average pore diameter (nm)	Pore volume (mL/g)
**P-1**	32.4	20.6	0.17
**P-2**	60.2	9.7	0.15
**P-3**	55.1	5.8	0.06
**C-1**	19.6	29.0	0.14
**C-2**	34.6	14.2	0.12
**C-3**	30.0	11.6	0.09

After the precursors was promoted, the specific surface area and pore volume of C-1, C-2 and C-3 are decreased except the pore volume of C-3. There are two possible factors responsible for the changes. Water, used as solvent in the impregnation of promoters, is the first one. Because water has high surface tension, pore structure, especially in small diameter, is destroyed when intrapore water is removed by drying [34]. It makes the loss of catalyst surface area. The second one is the distribution of promoter K in the catalysts. The radius of K^1+^ (1.33 Å) is two times of Fe^3+^ (0.64 Å). The impregnated K mainly distributes on the catalyst surface. It shrinks pore mouth, and even blocks off minor pores in catalyst. Thus, N_2_ molecule is prevented to enter into the inner of these pores in the experiment of N_2_ adsorption at low temperature. It results in small measured surface area and pore volume [32,35,36]. The morphologies of precursors and catalysts are shown in S1 Fig. There are large particles laying on C-1, which are confirmed to be K-containing particles by EDS (S2 Fig). The blocking to minor pores induces enlarged average pore diameter of the catalysts. S1 Fig also display that the surface of P-3 was fluffed after promoter impregnation, which is possible to induce abnormal increased pore volume of C-3.

### XPS characterization

**Fig 3:**
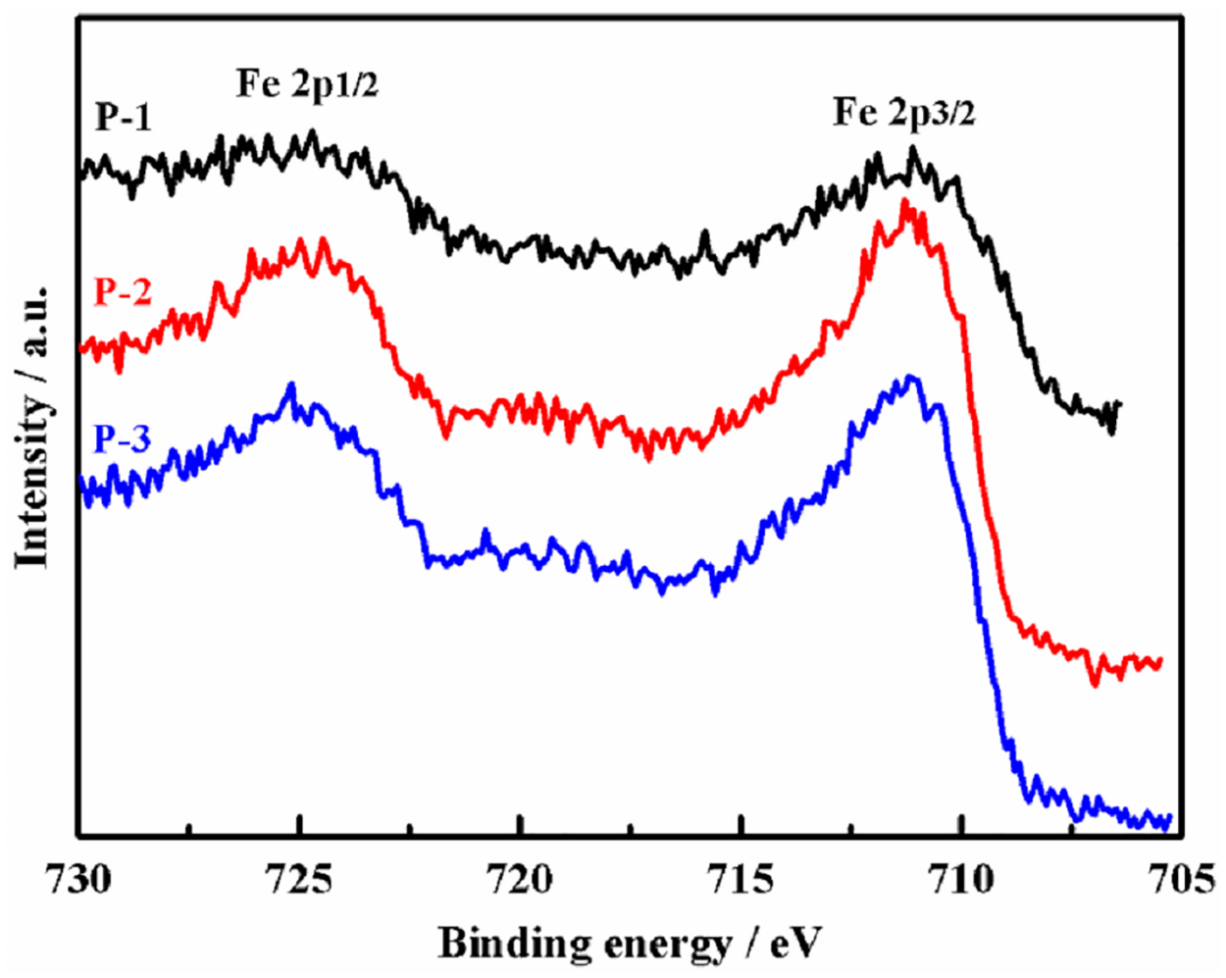
XPS spectrum of element Fe on the precursors.

Fig 3 gives the binding energy of Fe 2p in the precursors. The peaks of Fe 2p 3/2 and 2p 1/2 appears at 711.12 eV and 724.75 eV for the three precursors, respectively. The peak intensity of P-2 and P-3 is stronger than P-1, disclosing that the Fe quantity exposed on the surface of P-2 and P-3 is more than P-1. The deduction is supported by the results in Fig 1. The calculated particle size in P-1 is the largest among the three precursors. It results in the lowest surface content of Fe in P-1.

**Fig 4:**
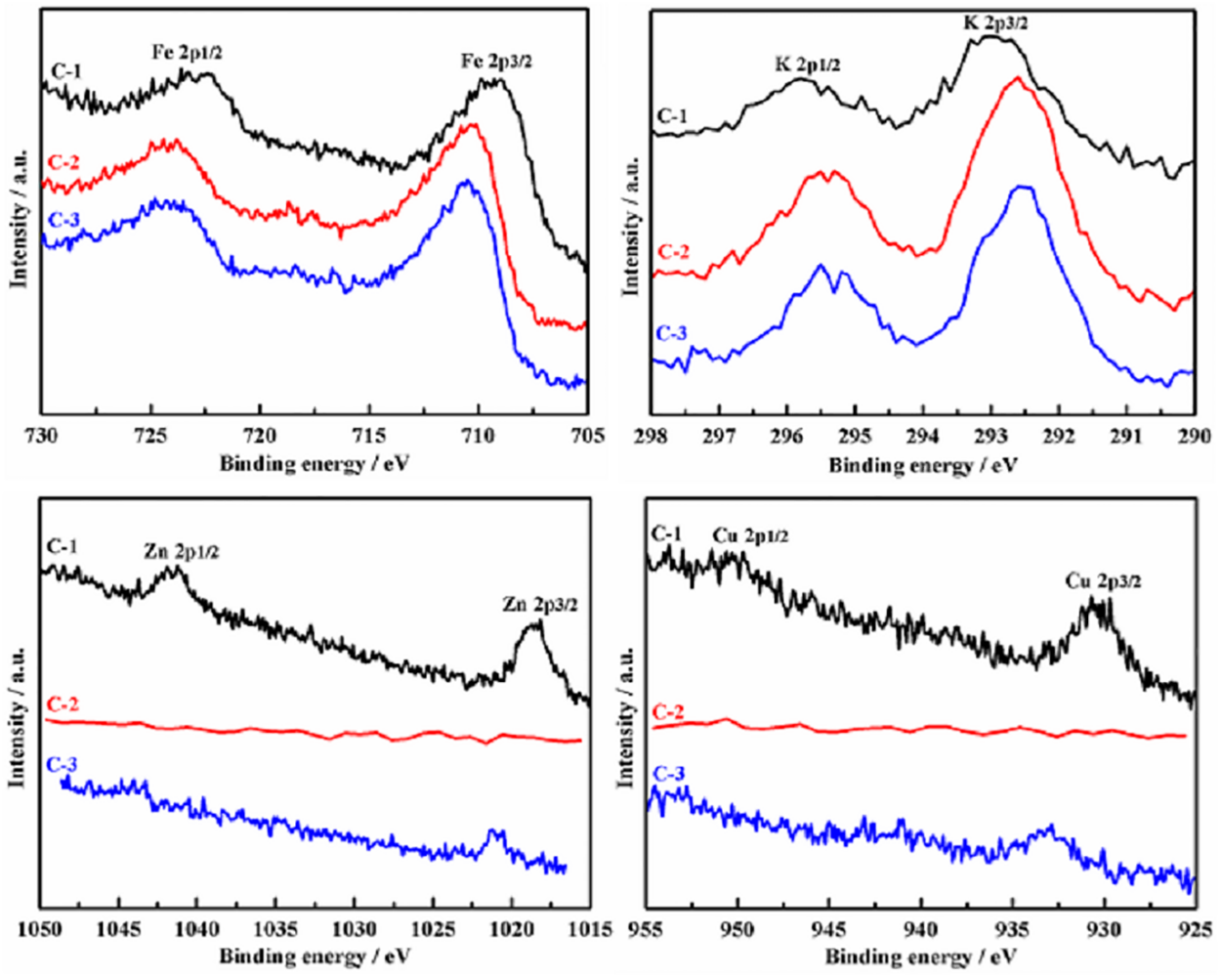
XPS spectra of element Fe, K, Zn and Cu on the catalysts.

The XPS results of catalyst C-1, C-2 and C-3 are shown in Fig 4. By comparing the peak area of element Fe, K, Cu and Zn, the dispersity of Fe and K on C-2 and C-3 surface is higher than C-1. On the contrary, Cu and Zn are more dispersive on C-1 than C-3. There is no signal of Cu and Zn for C-2. Promoter K mainly distributed on catalyst surface because of its large radius (1.33 Å for K^1+^ ion). From Table 1, the surface areas of C-2 and C-3 are bigger than C-1. It increases the ratio of Fe and K distributed on the catalyst surface. The radius of Zn^2+^ and Cu^2+^ is 0.74 Å and 0.72 Å, respectively. They are close to Fe^3+^ (0.64 Å). Therefore, promoter Zn and Cu can enter into the cation vacancy or replace Fe^3+^ in crystal Fe_2_O_3_. Because γ-Fe_2_O_3_ is more disorder than α-Fe_2_O_3_, it makes the inset of Cu and Zn into γ-Fe_2_O_3_ easier than α-Fe_2_O_3_. That is why the peak areas of Cu 2p and Zn 2p of C-3 are smaller than C-1. Due to the inserted Cu and Zn surrounded or interacted with much oxygen atom in C-3, their binding energies would be increased relative to C-1 which is reflected by the blue shift of Cu and Zn binding energy. C-2 is a mixture of γ-Fe_2_O_3_ and α-Fe_2_O_3_, and the mismatching between the two crystal phases produced much defective sites in C-2. Such disorder structure is beneficial to hold Cu and Zn in the bulk of catalysts. The assumption is supported by the observation of none of Cu 2p and Zn 2p signals for C-2 in Fig 4. Compared with the binding energy of Fe 2p 3/2 of the three precursors, the values of the three catalysts are decreased in the order of C-3 ≈ C-2 < C-1. Dry et al. [37] reported that K donates electrons to Fe. Therefore, a stronger electron shift from K to Fe happened in C-1 than C-2 and C-3.

### H_2_-TPR

**Fig 5:**
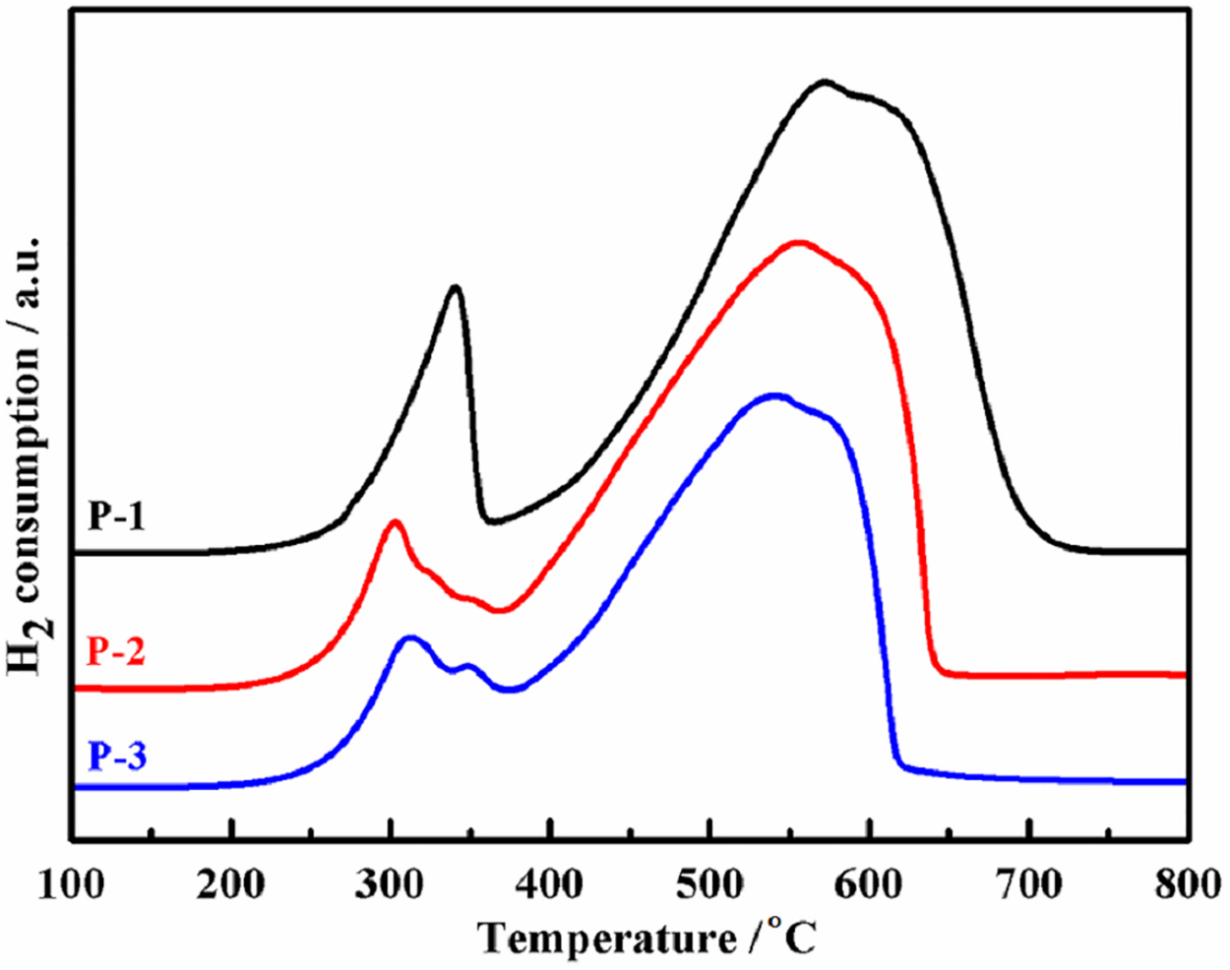
H_2_-TPR profiles of the precursors.

Fig 5 displays the reducibility of the three precursors. The peak temperature corresponding to Fe_2_O_3_ → Fe_3_O_4_ reduction is 340°C for P-1, while it is around 310°C for P-2 and P-3 [38,39]. Because γ-Fe_2_O_3_ is similar to Fe_3_O_4_ in view of their crystal structure [28,40], γ-Fe_2_O_3_ is more easily reduced to Fe_3_O_4_ than α-Fe_2_O_3_. The weak peak at 350°C in P-3 is the reduction of α-Fe_2_O_3_ which was produced from meso-stable γ-Fe_2_O_3_. The peak corresponding to the reduction of Fe_3_O_4_ → α-Fe is about 550°C for P-2 and P-3, but 574°C for P-1 [38,39].

**Fig 6:**
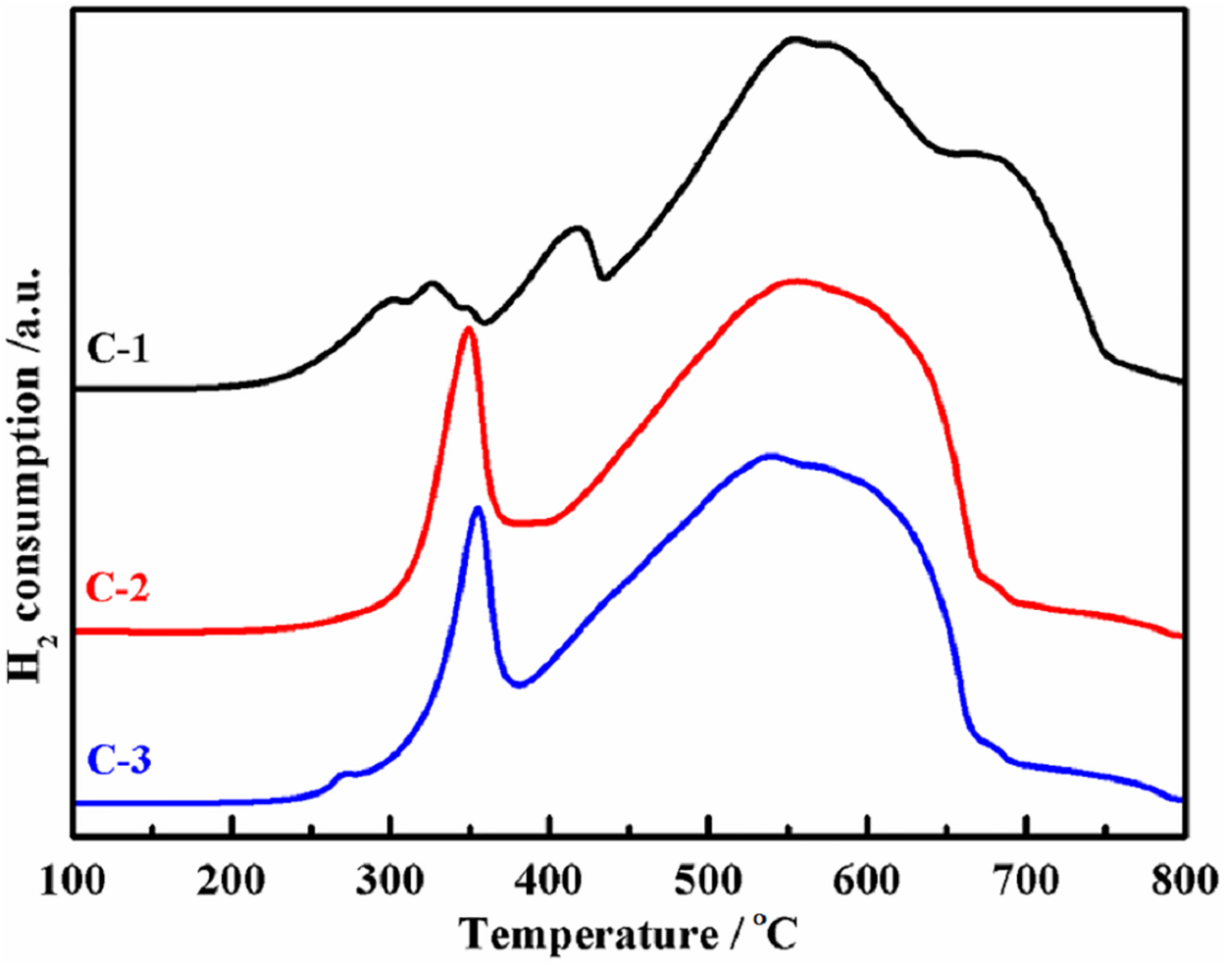
H_2_-TPR profiles of the catalysts.

Fig 6 presents the reducibility of the three catalysts. The wild peak below 350°C in C-1 is assigned to the reduction of CuO → Cu^0^ [32]. The data in Figs 2 and 4 disclose that Cu is highly dispersed into C-2 and C-3. Therefore, there is no evident reduction peak of CuO species for C-2 and C-3. A weak peak around 270°C appears in C-3 rather than C-2 is consistent with the observed Cu signal in C-3 and none in C-2 (Fig 4). The impregnated promoters usually induces shrunk surface area as shown in Table 1 [32,35,36]. It leads to decreased Fe dispersity and difficult reduction of iron oxides. Compared with the reduction behavior of the precursors in Fig 5, the corresponding process happened at higher temperature for the catalysts. For C-1, the H_2_-consumption peak at 420°C is from Fe_2_O_3_ → Fe_3_O_4_, and the wild peak centered at 570°C is assigned to Fe_3_O_4_ → α-Fe. The should peak around 680°C is the reduction of Fe_3_O_4_ covered by K-containing particles as shown in S1 Fig. K can inhibit the reduction of iron oxide by CO [41] and H_2_ [35]. The reduction process of iron oxides in C-2 and C-3 are almost same. The peak around 350°C are from highly dispersive Cu^2+^ → Cu^0^ and Fe_2_O_3_ → Fe_3_O_4_. The wide peak of H_2_ consumption in 370°C—660°C are from Fe_3_O_4_ → α-Fe. The peak temperature of C-2 and C-3 is about 558°C and 537°C, respectively. H_2_-TPR results disclose that C-2 and C-3 are easily reduced relative to C-1.

### Catalyst performance in CO_2_ hydrogenation

The influence of Fe_2_O_3_ crystal phases on CO_2_ hydrogenation is compared in Table 2. The catalytic activity (CO_2_ conversion) is proportional to the content of γ-Fe_2_O_3_ phase in the catalysts. C-3, composed of pure γ-Fe_2_O_3_ phase, is more active than C-1 which is composed of α-Fe_2_O_3_. Because C-2 contains α-Fe_2_O_3_ and γ-Fe_2_O_3_ simultaneously, its activity is between C-1 and C-3. The higher iron dispersity (Fig 4) and reducibility (Fig 6) of γ-Fe_2_O_3_ phase is beneficial to form more active site on C-2 and C-3 for CO_2_ hydrogenation than C-1, which results in the activity sequence of C-3 > C-2 > C-1.

**Table 2:** Reactive performance of the catalysts.

Catalyst	CO_2_ conv. (%)	Product distribution (C mol. %)			
		CO	CH_4_	C_2-4_	C_5_+
**C-1**	10.4	38.1	13.1	17.7	31.1
**C-2**	13.2	27.0	11.0	21.1	40.9
**C-3**	15.1	23.4	10.1	19.5	47.0

1.6 MPa, 230°C, 6 L/(h·g-cat.), H_2_/CO_2_ = 2

In view of CO selectivity, it declines with the increased content of γ-Fe_2_O_3_ phase in the catalysts. The selectivity of total hydrocarbons increases in the order of C-1 < C-2 < C-3. Especially, C-3 is the most active among the three catalysts to synthesize C_5_+ hydrocarbons. Promoter K is beneficial for CO_2_ hydrogenated into hydrocarbons [15] by inhibited H_2_ adsorption [20] and enhanced formation rates of C_2_+ hydrocarbons [42]. Although the nominal content of K is same for the three catalysts, Fig 4 unveils that the surface content of K on C-2 and C-3 is higher than C-1. The segregation of promoter K on C-2 and C-3 is responsible for the increased selectivity of hydrocarbons, because the surface atoms are the effective ones to influence the reaction.

## Conclusions

The crystal phase of Fe_2_O_3_ influences the catalyst reactivity in CO_2_ hydrogenation by two effects. The first effect is that the dispersity of both Fe and K on the catalyst in γ-Fe_2_O_3_ phase is higher than the catalyst in α-Fe_2_O_3_ phase. The second effect is that the catalyst in γ-Fe_2_O_3_ phase is more easily reduced than the one in α-Fe_2_O_3_ phase. The catalyst with high dispersive and easily reduced iron oxide can form much active site for CO_2_ hydrogenation and the high dispersive promoter K can increase the selectivity of C_2_+ hydrocarbons.

Aiming to reinforce the conclusion that γ-Fe_2_O_3_ phase is better than α-Fe_2_O_3_ phase to the Fe catalyst for CO_2_ hydrogenation, we are trying to prepare catalysts with similar specific surface area which are in α-Fe_2_O_3_ or γ-Fe_2_O_3_ phase, respectively. By studying such kind of catalysts, the effect of Fe_2_O_3_ crystal phases could be understand directly.

## Supporting information

S1 Fig The morphologies of precursors and catalysts observed by SEM. Every sample was observed under three levels of magnification.
(PDF)

S2 Fig Elements distribution on catalyst C-1 observed by EDS. (PDF)
