# Supplementary figures and images for: The effect of Fe2O3 crystal phases on CO2 hydrogenation
Source: PLoS One. 2017 Aug 14;12(8):e0182955. doi: 10.1371/journal.pone.0182955 (PMC5555619; doi:10.1371/journal.pone.0182955)

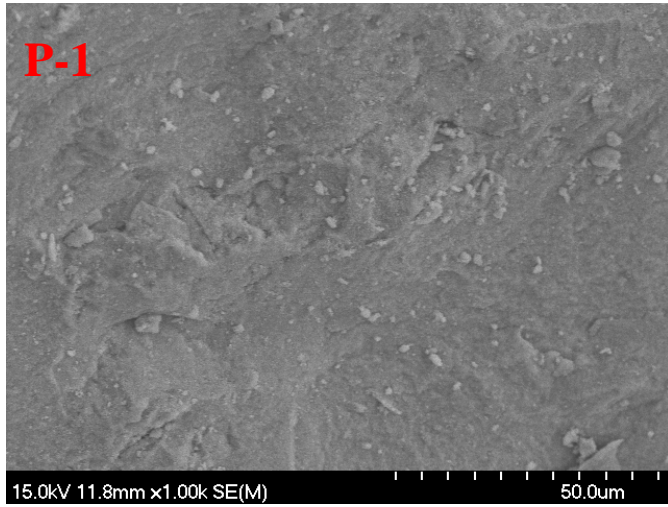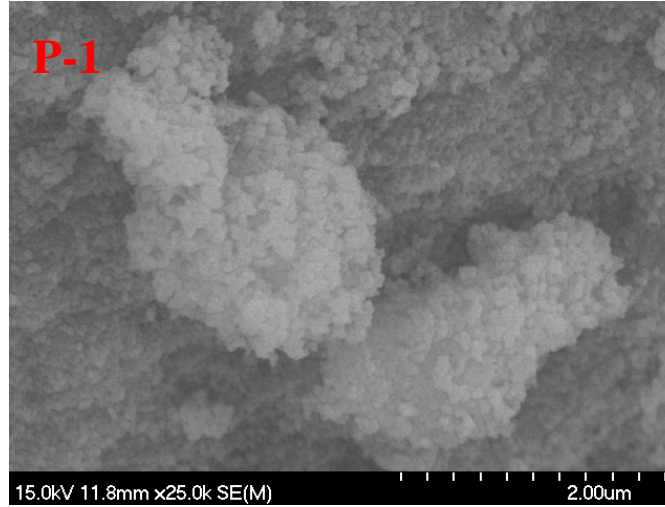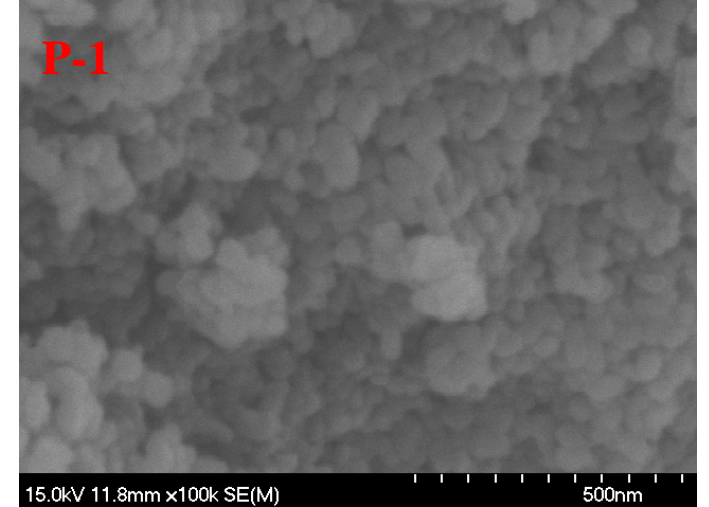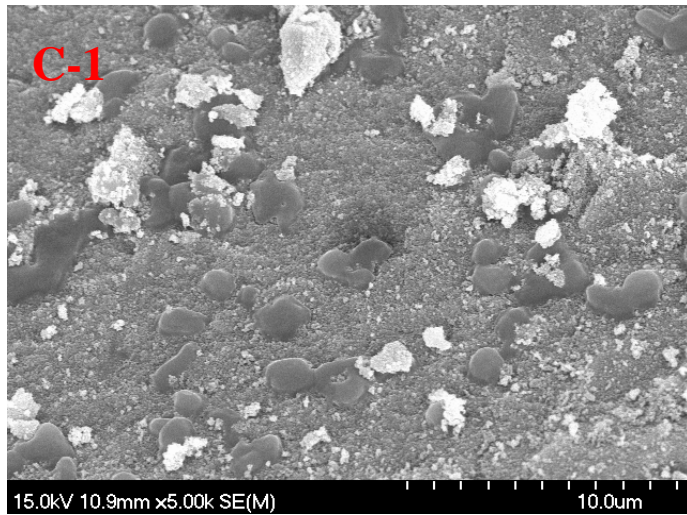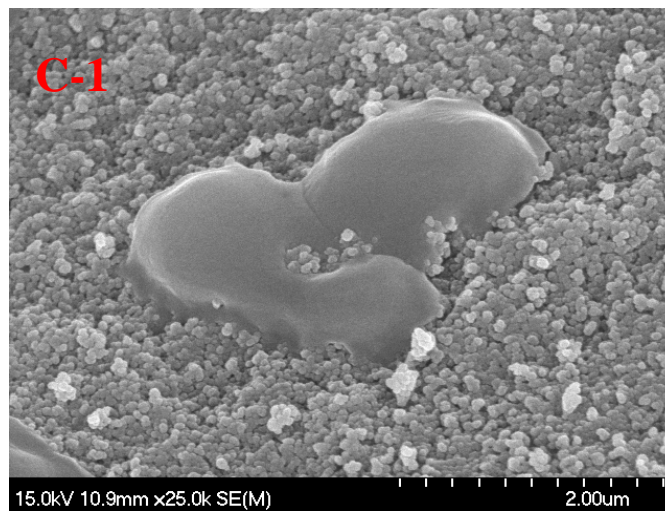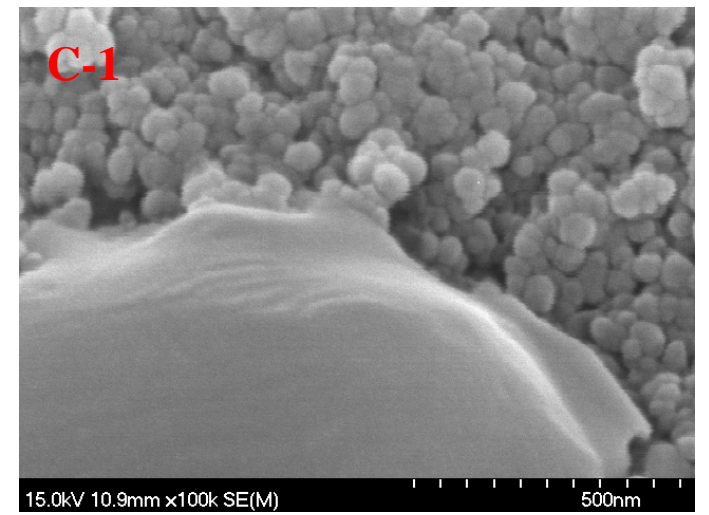

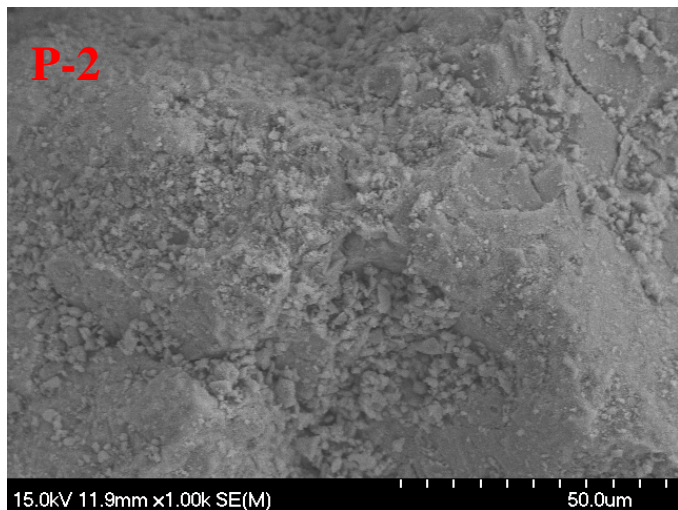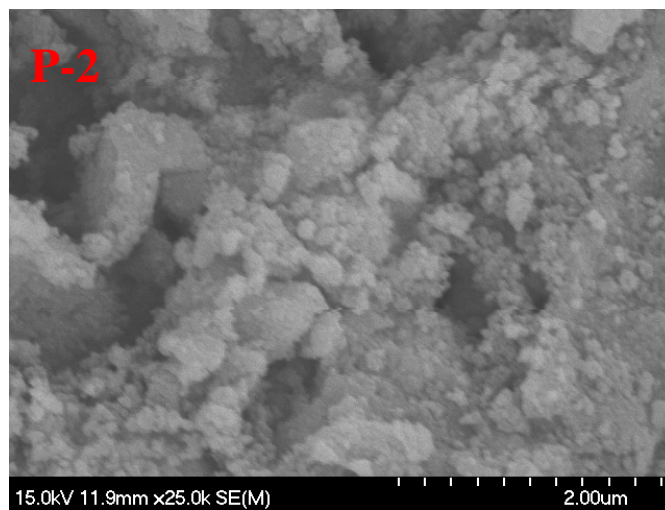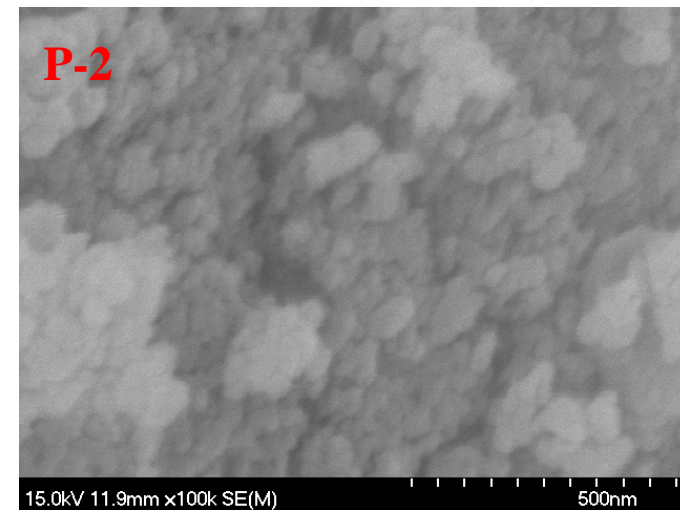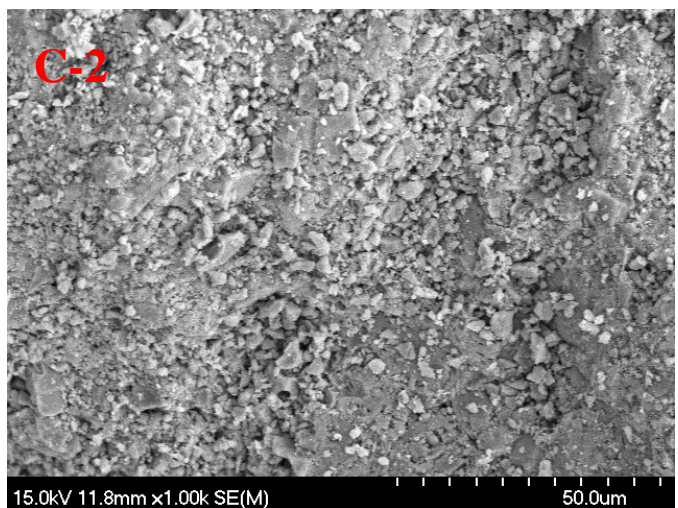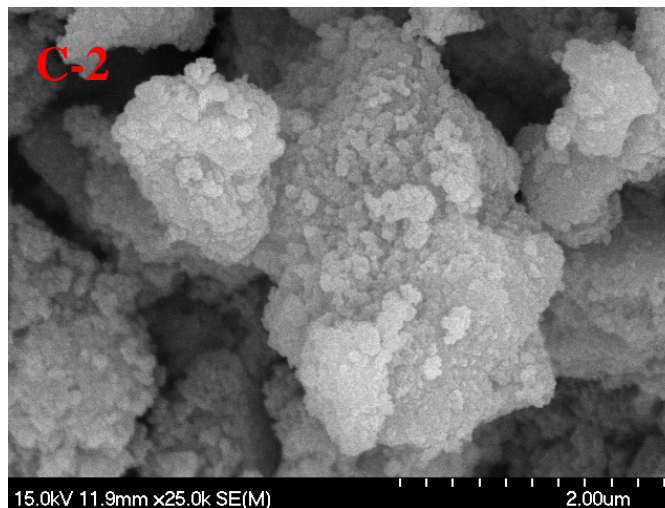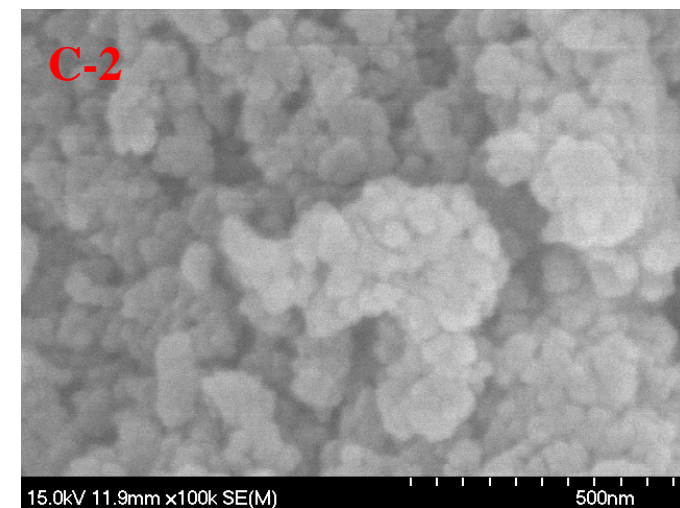

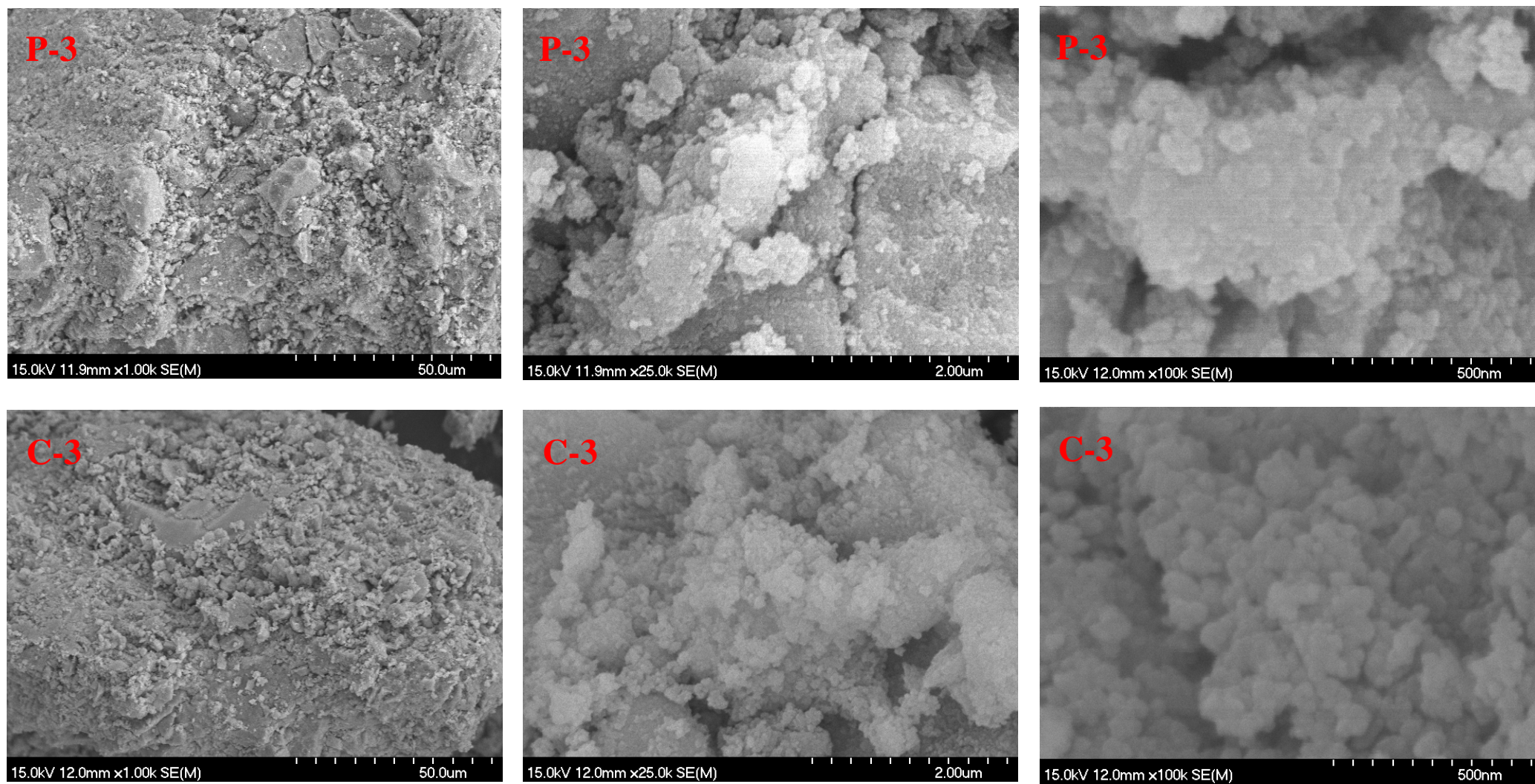

**S1 Fig.** The morphologies of precursors and catalysts observed by SEM.

Supplement: S1 Fig — Every sample was observed under three levels of magnification. (PDF) [file pone.0182955.s001.pdf]
