# Supplementary material for: The effect of Fe2O3 crystal phases on CO2 hydrogenation
Source: PLoS One. 2017 Aug 14;12(8):e0182955. doi: 10.1371/journal.pone.0182955 (PMC5555619; doi:10.1371/journal.pone.0182955)
Supplement: S2 Fig — (PDF) [file pone.0182955.s002.pdf]

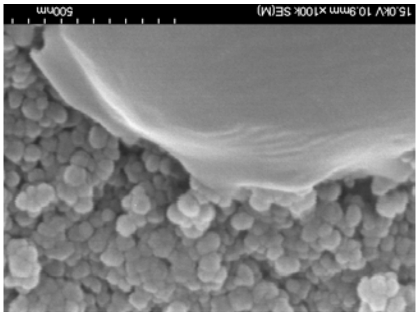

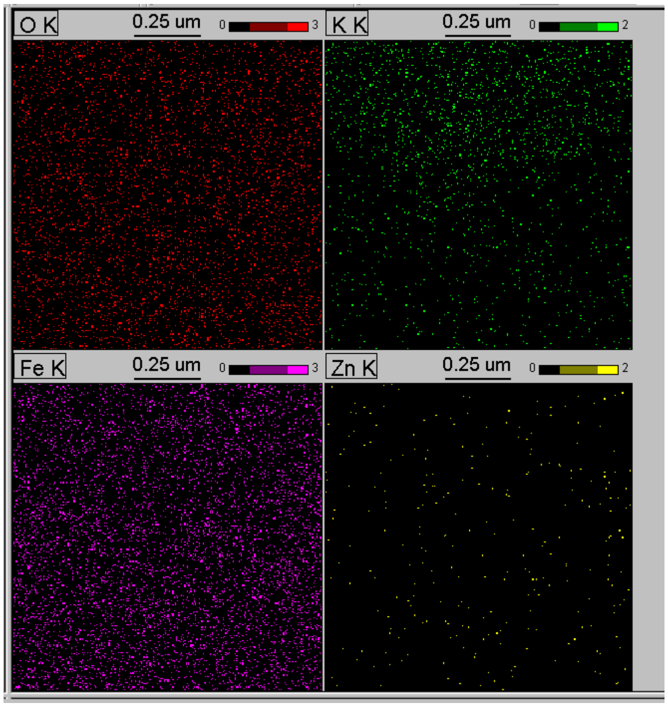

Element	k-ratio (calc.)	ZAF	Atom %	Element Wt %	Err. (1-Sigma)	No. of Cations
O -K	0.1531	1.802	56.61	27.59	+/- 0.77	---
K -K	0.0416	1.052	3.67	4.38	+/- 0.15	1.558
Fe-K	0.5988	1.082	38.10	64.81	+/- 0.97	16.152
Zn-K	0.0275	1.173	1.62	3.23	+/- 0.95	0.687
Total			100.00	100.00		18.397

The number of cation results are based upon 24 Oxygen atoms

S2 Fig. Elements distribution on catalyst C-1 observed by EDS.
